# Allergen sensitization pattern of allergic adults and children in southern China: a survey based on real life data

**DOI:** 10.1186/s13223-019-0357-y

**Published:** 2019-07-24

**Authors:** Wenting Luo, Haisheng Hu, Wangbing Tang, Xiangwei Zou, Huimin Huang, Zhifeng Huang, Yong Liu, Baoqing Sun

**Affiliations:** 1 Department of Allergy and Clinical Immunology, State Key Laboratory of Respiratory Disease, National Clinical Research Center of Respiratory Disease, Guangzhou Institute of Respiratory Health, First Affiliated Hospital of Guangzhou Medical University, Guangzhou Medical University, 151 Yanjiangxi Road, Guangzhou, 510120 Guangdong China; 2 Longgang District People’s Hospital, Shenzhen, 518172 China; 3 Guangzhou Kingmed Diagnostics Group Co., Ltd., Guangzhou, 510120 China

**Keywords:** Southern China, Allergen sIgE, Cockroach, Multiple sensitization

## Abstract

**Background:**

Allergic diseases are increasing yearly. We aimed to evaluate the difference in allergen sensitization pattern between adults and children in southern China by analyzing a large sample size of real-life data, and to provide the evidence for formulating the prevention and management strategies.

**Methods:**

Retrospective analysis was conducted on 39,813 serum allergen-specific IgE (sIgE) results collected in southern China from January to December in 2017. Sensitization patterns and how these allergens could lead to the allergic diseases were analyzed for adults and children respectively. The difference of allergen positive rate between groups was calculated using the Chi square test.

**Results:**

The top five most sensitized allergens in southern China were house dust mite (28.1%), cockroach (24.3%), shrimp (19.2%), crab (15.5%) and egg white (9.9%). While cockroach had the highest positive rate in adults (29.2%), the most sensitized allergen in children was house dust mite (29.7%). The positive rates of egg white and cow’s milk in children were higher than in adults for the whole year (p < 0.001); whereas for adults, the positive rate to cockroach, shrimp and crab were higher than in children (p < 0.001). The positive rate of house dust mite in coastal cities (32.4%) was higher than that in landlocked cities (24.0%) in children, but in adults, the rate in landlocked cities (31.1%) was higher than that in coastal cities (25.3%). Optimal scaling analysis showed that the sIgEs of crab and shrimp had the closest correlation with cockroach-sIgE (Cronbach’s α = 0.891). The positive rate to mold allergens increased from summer to autumn, reaching its plateau in October (6.2%). Patients with skin diseases were found to receive the highest sIgE prescription from doctors (56.9%).

**Conclusions:**

Doctors tend to prescribe more sIgE tests for patients with skin disorders in southern China. In addition to house dust mite, cockroach was found to be another important allergen. Adults with multiple sensitization always showed co-sensitization with shrimp, crab and house dust mite. These should be taken into consideration when giving allergen avoidance advice to patients.

**Electronic supplementary material:**

The online version of this article (10.1186/s13223-019-0357-y) contains supplementary material, which is available to authorized users.

## Background

Allergic diseases affect 22% of the world’s population [1], and create significant healthcare burden to the community and affect the quality of life of the patient. Treatment and prevention of the diseases rely on correct identification of the causative allergens [2] and implementation of various avoidance measures.

Allergens can be divided into inhalant and food. The most common inhalant allergens found in the western studies include house dust mite, mold, cockroach and animal dander whereas peanut, cow’s milk, egg, crab and shrimp were identified as the most common food allergens [3]. The major in vitro method to identify the causative allergens is serum allergen specific Immunoglobulin E (sIgE) test. ImmunoCAP (ThermoFisher, USA) is the most widely used sIgE test around the world and is routinely used for clinical diagnosis and research purpose [4, 5].

Southern China refers mainly to the area to the south of Qinling and Huaihe (103°E–123°E, 22°N–34°N), surrounded in the west, east, and south by Qinghai–Tibet Plateau, the East China Sea, and the South China Sea, respectively. The whole region accommodates 55% of the population of China (about 740 million). Due to its big geographic coverage and variation in weather, the analysis of allergen that triggers allergy is very complicated [6]. A comprehensive and correct choice of allergen panel is of prime importance to aid diagnosis. Moreover, owing to the modernization of the country, the lifestyle of people has also changed tremendously. All these factors can trigger the continuous change in people’s susceptibility to various allergens [7] and hence a regular evaluation of allergen distribution throughout different seasons among different age groups is necessary [8]. Nevertheless, such studies on adults and children in southern China based on real-life medical data are scarce [9].

KingMed Diagnostics Center (KMD) (http://en.kingmed.com.cn) is a commercial laboratory in China with ISO/IEC17025; ISO9001; ISO15189 accreditation and is recognized by College of American Pathologists [10]. With its headquarters located in Guangzhou city, KMD offers laboratory testing services to more than 29 provinces and cities in southern China including Guangzhou, Kumming and Shanghai, et al. This study aimed to investigate the distribution of allergen sensitization pattern among adults and children in southern China by analyzing the real-life data from the KMD. The results of our study can serve as an objective reference for allergen prevalence, not only for the studied areas, but possibly for other developing countries in the world with similar climatic and geographical conditions. It can provide guidance for allergen avoidance measures and evidence-based allergy prevention.

## Methods

### Study design and objective

Approval was obtained from the ethic committee of The First Affiliated Hospital of Guangzhou Medical University (Reference number: GYFYY-2016-73).

The analysis was based on the data extracted from the KMD’s database for sIgE determination with ImmuoCAP (ThermoFisher, USA) from January 1, 2017 to December 31, 2017. Patients with cancer, immunodeficiency, parasitic infection, autoimmune diseases and those with no data regarding age were excluded, and the remaining patients were enrolled in this study.

**Fig. 1:**
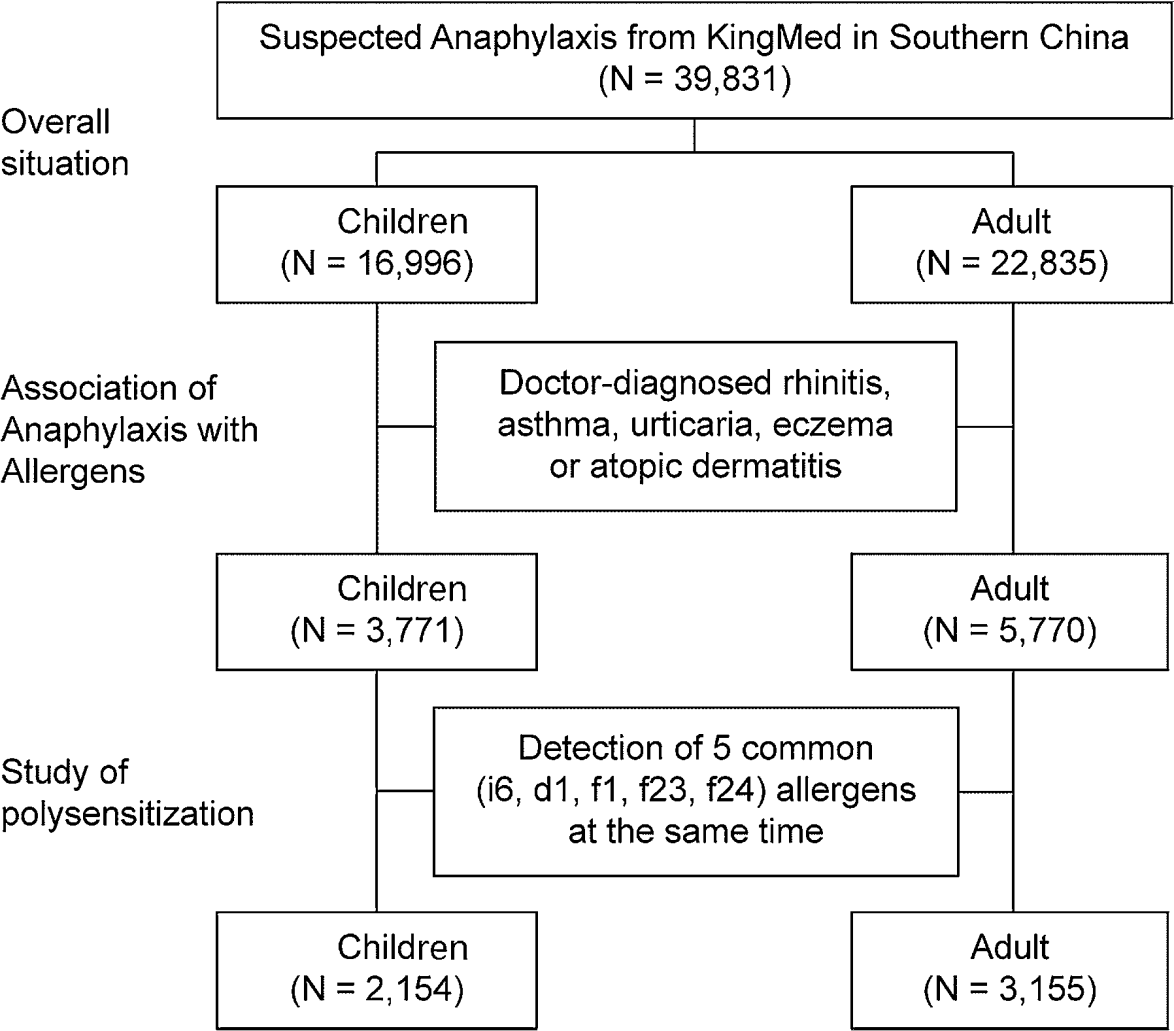
Study design. A total of 39,831 patients from southern China were enrolled in the study, including 22,835 adults and 16,996 children. Among them, 9541 patients were diagnosed by doctors as having rhinitis, asthma, urticaria, eczema or atopic dermatitis. Of these, 5309 patients were also tested for i6: cockroach, d1: house dust mite, f2: cow’s milk, f23: crab, f24: shrimp

The following symptoms were suspected to have been a cause for allergy by the doctor: dyspnea, wheezing and/or cough not attributable to common cold, runny nose, sneezing, nasal itching/obstruction, rashes, wheal, urticaria, abdominal pain, diarrhea, indigestion and itchy eyes; serum sIgE tests were ordered to confirm causative allergen(s). The resulted sensitization patterns were analyzed to identify the seasonal and geographical difference as well as their correlation to various symptoms among adults and children (Fig. 1).

While KMD’s allergen panel consisted of 20 allergens, the results of the following nine allergens, which were prescribed for more than 3000 times in this year, were selected for the analysis: Mold (Mold mix 1: *Penicillium chrysogenum, Cladosporium herbarum, Aspergillus fumigates* and *Alternaria alternata*), cockroach (*Blattella germanica*), house dust mite (*Dermatophagoides pteronyssinus*), egg white (*Gallus domesticus*), cow’s milk (*Bos domesticus*), peanut (*Arachis hypogaea*), crab (*Cancer pagurus*), shrimp (*Penaeus monodon*) and dog dander (*Canis familiaris*). The impact of small sample size by other allergens was discussed in Additional file 1: Table S1.

### Transportation of samples and detection method

5 mL venous blood sample were taken from the hospital’s clinic with separation gel vacuum coagulation tube, and was centrifuged for 10 min at 1006.2×*g* and recovered for the serum supernatant. They were then cold-chain delivered to KMD centers in their respective area. Sera were analyzed using ImmunoCAP 1000 (ThermoFisher, USA) by trained technicians and the result was presented as kU/L, with ≥ 0.35 kU/L as positive cutoff.

### Statistical method

Data was analyzed using SPSS 22.0 (IBM Corp, Armmonk, NY, USA). Normal distribution data such as age were presented as mean + standard error. Non-normally distributed data such as concentration of sIgE were presented as median (interquartile intervals 25%, 75%). Qualitative information such as positive rate was presented as percentage or frequency. Inter-group difference was calculated using the Chi square test (χ^2^). Interrelationship between allergens was analyzed using optimal scale analysis, and statistical significance was defined as p < 0.05.

## Results

### Totality

A total of 39,831 patients aged 25.63 ± 21.34 years were enrolled in this study. Among them, 22,835 were adults (aged ≥ 18 years) and 16,996 were children (aged < 18 years); 12,348 were from inland cities (including Sichuan, Chongqing, Hunan, Guizhou) and 27,483 were from coastal cities (including Guangdong, Guangxi, Hainan, Yunnan, Fujian, Shanghai).

Among the 39,831 patients, 14,862 (37.3%) were found to have one or many of following symptoms: dyspnea, wheezing and/or cough not attributable to common cold, runny nose, sneezing, nasal itching/obstruction, rashes, wheal, urticaria, abdominal pain, diarrhea, indigestion and itchy eyes. Among this subgroup, 8455/14,862 (56.9%) were confirmed to have urticaria, eczema and/or atopic dermatitis and 1086/14,862 (7.3%) had respiratory diseases such as asthma and/or rhinitis.

Among the 39,831 samples, the most common sensitized allergens were house dust mite (28.1%), cockroach (24.3%), shrimp (19.2%), crab (15.8%) and egg white (9.9%), with cockroach and house dust mite as the most common allergens in adults (29.2%) and children (29.7%) respectively. The sensitization to egg white (20.3% vs. 1.7%, χ^2^ = 1930.82, p < 0.001) and cow’s milk (20.3% vs. 1.2%, χ^2^ = 2139.41, p < 0.001) were higher in children than in adults (Table 1).

**Table 1:** Baseline characteristics of 39,831 patients from southern China

Characteristic	Total	Adult	Children	*p*
Total (N)	39,831	22,835	16,996	–
Sex (male/female)	18,345/21,360	8362/14,395	9983/6965	< 0.001*
Age (mean ± SD)	26 ± 21	41 ± 15	5 ± 4	–
Season (N)				
Spring (3–5)	8393	4667	3726	< 0.001*
Summer (6–8)	12,863	7288	5575	
Autumn (9–11)	10,351	6034	4317	
Winter (12–2)	8219	4844	3375	
Disease (N)				
Rhinitis	782	339	443	< 0.001*
Asthma	304	145	159	
Urticaria	4151	2635	1516	
Eczema	858	573	285	
Atopic dermatitis	3446	2078	1368	
Positive for allergen (a/n, %)				
Mold	590/18,317 (3.2)	316/10,589 (3.0)	274/7728 (3.5)	0.034*
*Blattella germanica*	4545/18,668 (24.3)	3059/10,476 (29.2)	1486/8192 (18.1)	< 0.001*
*Dermatophagoides pteronyssinus*	6013/21,411 (28.1)	3392/12,605 (27.3)	2621/8806 (29.7)	0.002*
*Gallus domesticus*	1993/20,216 (9.9)	194/11,343 (1.7)	1799/8873 (20.3)	< 0.001*
*Bos domesticus*	1953/20,416 (9.6)	132/11,463 (1.2)	1821/8953 (20.3)	< 0.001*
*Arachis hypogaea*	209/4031 (5.2)	95/1990(4.8)	114/2041 (5.6)	0.245
*Cancer pagurus*	3173/20,125 (15.8)	2157/11,664 (18.5)	1016/8461 (12.0)	< 0.001*
*Penaeus monodon*	3879/20,228 (19.2)	2645/11,500 (23.0)	1234/8728 (14.1)	< 0.001*
*Canis familiaris*	679/20,158 (3.4)	385/11,491 (3.4)	294/8667 (3.4)	0.871

*n* test number, *a* positive number

* Chi square tests, *p* < 0.05. Mold (Mold mix 1: *Penicillium chrysogenum, Cladosporium herbarum, Aspergillus fumigates* and *Alternaria alternata*)

### Monthly distribution of allergen sensitization in adults and children

**Fig. 2:**
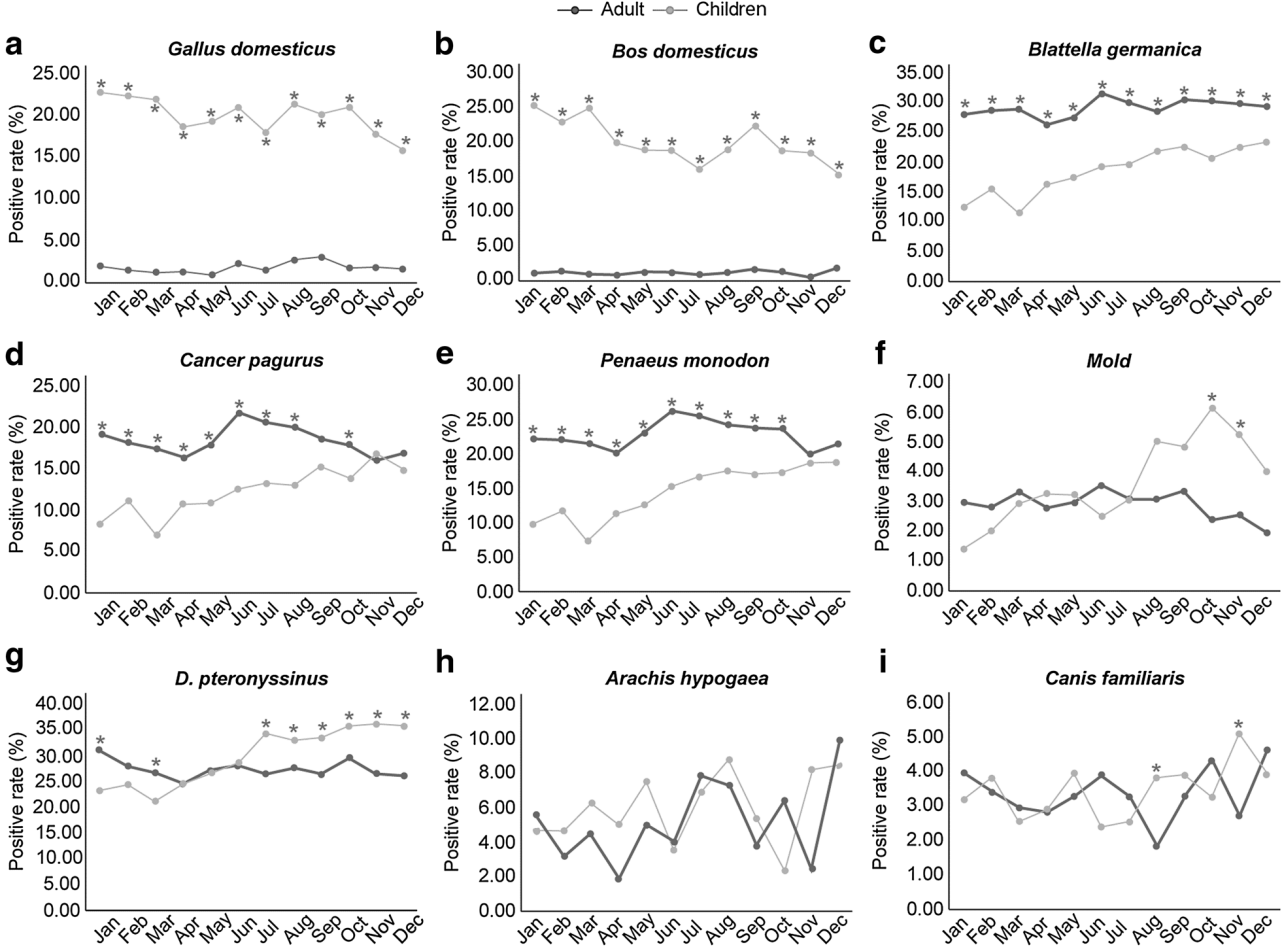
The distribution of allergen-positive rates between children and adults according to different seasons. *Chi square test, *p* < 0.05. The allergen-positive rate between children and adults for **a** *Gallus domesticus:* egg white; **b** *Bos domesticus:* cow’s milk; **c** *Blattella germanica*: cockroach; **d** *Cancer pagurus*: crab; **e** *Penaeus monodon*: shrimp; **f** Mold (Mold mix 1*: Penicillium chrysogenum, Cladosporium herbarum, Aspergillus fumigates and Alternaria alternata*); **g** *Dermatophagoides pteronyssinus*: house dust mite, **h** *Arachis hypogaea:* peanut; and **i** *Canis familiaris:* dog dander

The positive rates to egg white and cow’s milk in adults were lower than 3% for the whole year, and were significantly lower than those in children (p < 0.05) (Fig. 2a, b). However, the opposite trend was seen for cockroach, shrimp and crab, with children having a consistently lower positive rate (p < 0.05) (Fig. 2c–e). The positive rate to mold in children increased from summer to autumn and reached its maximum (6.16%) in October. It was significantly higher than adult levels in autumn (5.4% vs. 2.8%, χ^2^ = 17.12, p < 0.001) (Fig. 2f). In contrast, the positive rate to house dust mite from July was significantly higher in children than in adults (35.2% vs. 26.8%, χ^2^ = 52.07, p < 0.001) while from January to March, it was lower than that in adults (23.4% vs. 27.9%, χ^2^ = 14.13, p < 0.001) (Fig. 2g). No seasonal variation was seen in peanut and dog dander (Fig. 2h, i).

### Geographical distribution of allergen sensitization in adults and children

**Fig. 3:**
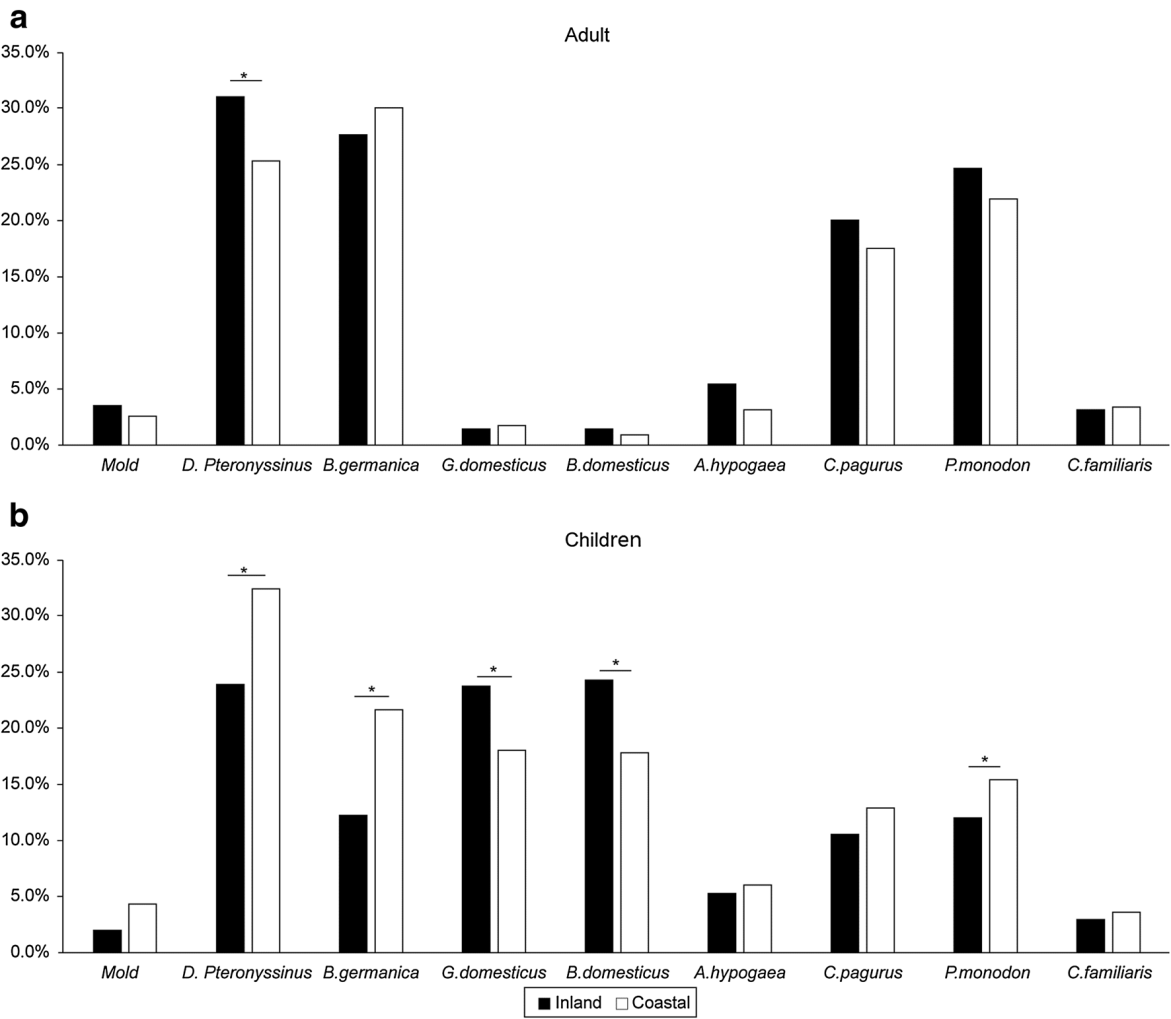
**a** The distribution of allergen-positive rate between inland and coastal city in adult. **b** The different of allergen-positive rate between inland and coastal city in children. *Chi square test, *p* < 0.05

In adults, the positive rate to cockroach was the highest in coastal cities (30.5%) and that to house dust mite was the highest in inland cities (31.1%). The positive rate of house dust mite in inland cities (31.1%) were higher than that in coastal cities (25.3%, p < 0.001) (Fig. 3a). In children, the positive rate to house dust mite was the highest both in coastal cities (32.4%) and inland cities (24.0%). The positive rate to egg white (23.8% vs. 18.1%) and cow’s milk (24.3% vs. 17.9%) allergen in inland cities were higher than in coastal cities (p < 0.001), but that to cockroach (21.6% vs. 12.3%) were higher in coastal cities than in inland cities (p < 0.001) (Fig. 3b).

### Inter-disease distribution of allergen sensitization in adults and children

House dust mite was the major sensitized allergen in children with asthma (55.3%), rhinitis (29.5%), urticaria (27.5%) and atopic dermatitis (28.4%). Among the children with eczema, cow’s milk (27.8%) and egg white (24.7%) had the highest positive rate. In adults, cockroach was the major allergen for asthma (36.8%), urticaria (34.0%), rhinitis (33.9%), atopic dermatitis (29.3%) and eczema (25.7%). For adults with urticaria, the highest positive allergens were cockroach (34.0%, p < 0.05), house dust mite (28.1%, p < 0.05) and shrimp (25.4%, p < 0.05) (Table 2). There was no statistically significant difference in the positive rate to dog dander and peanut in patients with all diseases. Among the 693 patients who were multi-sensitized to crab, shrimp, cockroach, and house dust mite, 48.8% had eczema, 33.2% had atopic dermatitis, 9.7% had rhinitis, 5.3% had eczema, and 3.0% had asthma.

**Table 2:** Distribution of allergen-positive rates between children and adults according to different diseases

Characteristic	Asthma	Rhinitis	Urticaria	Atopic dermatitis	Eczema	χ^2^	*p*
Adult positive (a/n, %)							
Mold	11/99 (11.1)	8/225 (3.6)	32/1505 (2.1)	21/1138 (1.8)	17/360 (4.7)	38.28	< 0.001*
*Blattella germanica*	35/95 (36.8)	74/218 (33.9)	481/1415 (34.0)	330/1127 (29.3)	87/339 (25.7)	*13.85*	0.008*
*D. pteronyssinus*	21/95 (22.1)	60/222 (27.0)	409/1456 (28.1)	292/1164 (25.1)	67/348 (19.3)	*12.93*	0.012*
*Gallus domesticus*	1/93 (1.1)	2/222 (0.9)	36/1495 (2.4)	18/1161 (1.6)	11/348 (3.2)	*6.44*	*0.169*
*Bos domesticus*	0/93	1/222 (0.5)	17/1494 (1.1)	5/1161 (0.4)	5/349 (1.4)	*6.56*	*0.161*
*Arachis hypogaea*	0/8	11/339 (3.2)	68/2635 (2.6)	1/78 (1.3)	1/14 (7.1)	*2.40*	*0.662*
*Cancer pagurus*	19/97 (19.6)	43/232 (18.5)	341/1626 (21.0)	219/1228 (17.8)	48/360 (13.3)	*12.79*	0.012*
*Penaeus monodon*	23/95 (24.2)	51/223 (22.9)	372/1463 (25.4)	259/1161 (22.3)	63/350 (18.0)	*9.90*	0.042*
*Canis familiaris*	5/95 (5.3)	9/221 (4.1)	45/1455 (3.1)	33/1157 (2.9)	8/348 (2.3)	*3.18*	*0.528*
Children positive (a/n, %)							
Mold	9/101 (8.9)	11/292 (3.8)	28/889 (3.1)	22/803 (2.7)	2/124 (1.6)	*12.26*	0.016*
*Blattella germanica*	29/113 (25.7)	83/297 (27.9)	187/864 (21.6)	176/839 (21.0)	9/126 (7.1)	*23.90*	< 0.001*
*Dermatophagoides pteronyssinus*	63/114 (55.3)	90/305 (29.5)	250/908 (27.5)	246/865 (28.4)	30/147 (20.4)	*44.55*	< 0.001*
*Gallus domesticus*	32/114 (28.1)	32/306 (10.5)	190/925 (20.5)	154/873 (17.6)	44/158 (27.8)	*31.09*	< 0.001*
*Bos domesticus*	21/114 (18.4)	30/309 (9.7)	165/926 (17.8)	153/874 (17.5)	40/162 (24.7)	*19.03*	0.001*
*Arachis hypogaea*	2/17 (11.8)	2/16 (12.5)	1/57 (1.8)	6/113 (5.3)	3/43 (7.0)	*4.36*	*0.360*
*Cancer pagurus*	18/104 (17.3)	46/311 (14.8)	133/942 (14.1)	108/865 (12.5)	7/139 (5.0)	*11.37*	0.023*
*Penaeus monodon*	25/112 (22.3)	54/306 (17.6)	148/910 (16.3)	126/866 (14.5)	9/146 (6.2)	*15.80*	0.003*
*Canis familiaris*	10/112 (8.9)	10/302 (3.3)	23/904 (2.5)	33/860 (3.8)	2/141 (1.4)	14.78	0.005*

*n* test number, *a* positive number

* Chi square tests, *p* < 0.05. Mold (Mold mix 1: *Penicillium chrysogenum, Cladosporium herbarum, Aspergillus fumigates* and *Alternaria alternata*)

### Adults and children with multisensitization

**Fig. 4:**
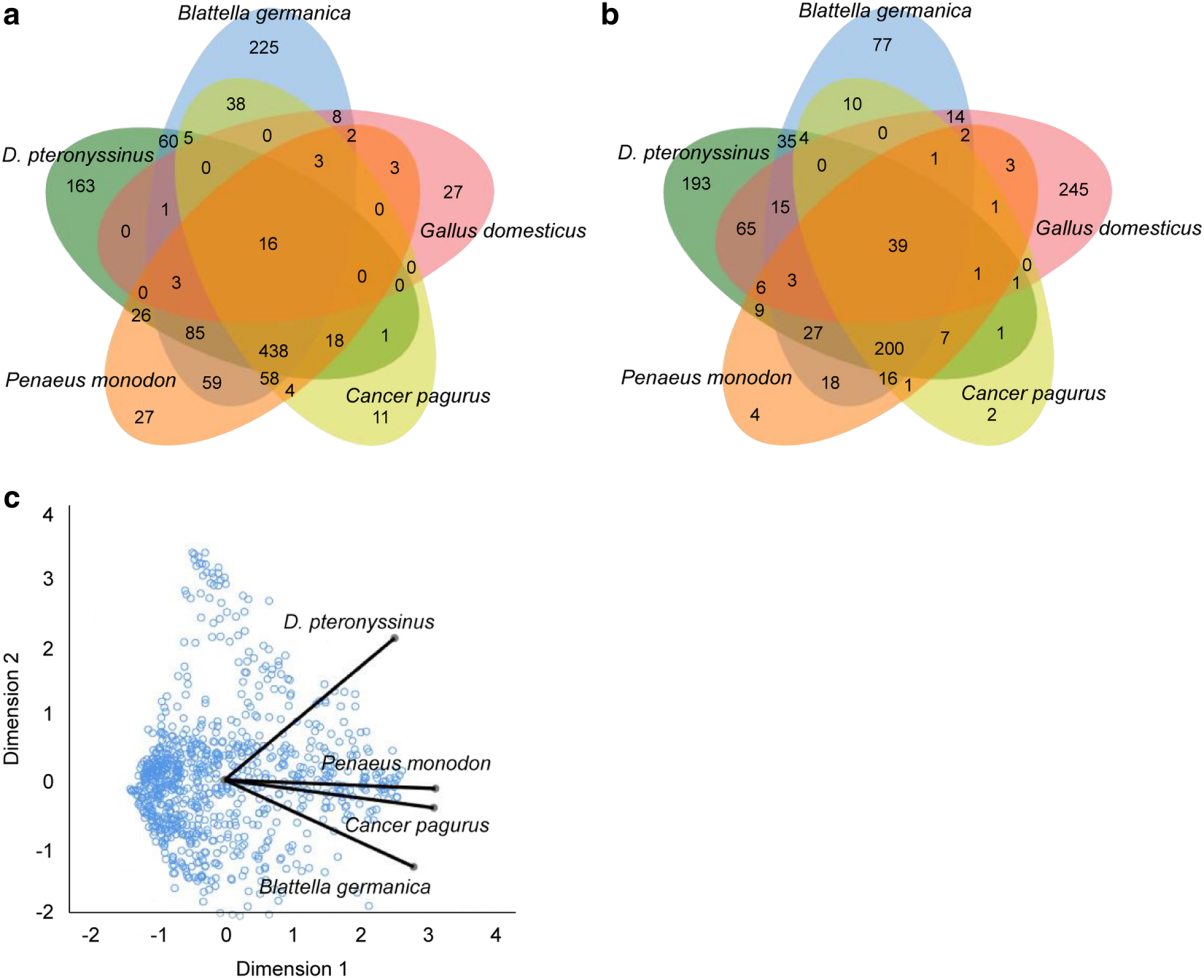
Polysensitization in children and adults. A total of **a** 3155 adult patients and **b** 2154 children were also tested for *Dermatophagoides pteronyssinus*: house dust mite, *Bos domesticus*: cockroach, *Cancer pagurus*: crab, *Penaeus monodon*: shrimp, and *Blattella germanica:* cockroach. The figure shows the number of allergen-positive patients. **c** The distance between the points represents the relative strength of correlation between them, the shorter the distance between the two points, the higher the correlation. The optimal scale analysis shows that there is a close relationship between cockroach, crab and shrimp (Cronbach’s α = 0.891)

SIgE test for five allergens including house dust mite, cow’s milk, crab, shrimp, and cockroach were performed for 5309 patients with rhinitis, asthma, urticaria, atopic dermatitis, and eczema. Among them, 40.0% (2281) were sensitive to at least one allergen, with children and adults accounting for 1000 (46.4%) and 1281 (40.6%) respectively. While most of the patients, irrespective of whether adults or children, showed sensitization to house dust mite, cockroach, shrimp, and crab, 92.5% (810/876) of patients with sensitization to crab also had sensitization to cockroach and/or house dust mite, and only 1.5% (13/876) were mono-sensitive to crab. Further analysis on the crab sensitive patients showed that its co-sensitization rate to only cockroach, shrimp, or house dust mite were just 5.5% (48/876), 0.5% (4/876) and 0.2% (2/876). Of the patients, 72.9% (638/876) were co-sensitive to all the three allergens. Moreover, among those co-sensitive to crab and shrimp, 99.4% (798/803) were positive for inhalant allergens while in patients only sensitive to crab, the rate was lower, at 82.2% (60/73). This was in sharp contrast to the 7.1% (62/876) [adults: 2.2% (19/876), children: 5.0% (43/876)] co-sensitization rate between crab and egg white (Fig. 4a, b). Optimal scaling analysis also showed cockroach had a closer correlation to crab and shrimp than to house dust mite (Cronbach’s α = 0.891) (Fig. 4c).

## Discussion

It is well accepted that pro-active disease prevention is the most effective and economical way for improving the health of humans, and hence, studies of actual allergen sensitization patterns can help provide direction for these efforts [10]. The current study retrospectively analyzed sensitization patterns of 39,831 patients from southern China and found that house dust mite (28.1%) and cockroach (24.3%) were the most important inhalant allergens in the region. The most important food allergens were shrimp (19.2%) and crab (15.8%).

House dust mite is perceived as the most important inhalant allergen in southern China [11]. In the current study, the positive rate to house dust mites was found to be lower than previous studies. Such discrepancy might be attributable to differences in subject recruitment. The current study was the first one conducted in southern China which is based on the real-life medical data. In contrast to other previous epidemiological studies that focused on patients with a history of respiratory allergies such as asthma and rhinitis [12], our patients were recruited from various basic medical centers and hospitals where doctors prescribed sIgE tests to those allergy suspects based on patient clinical history and symptoms. We covered a broader spectrum of patients including those with allergic skin diseases. In fact, our data also showed that doctors tended to prescribe more allergen tests to patients with skin problems (56.9%, 8455/14,862) (Table 1), which also explained why we have more data from skin disease patients than others. With the improvement in living standard and higher economic incomes, people are paying more attention to skin allergies and hence more education about impacts of various allergens on allergy prevention may be helpful. Nevertheless, the lower sIgE prescription from respiratory doctors might also indicate that the doctor worked at a basic medical center or hospital and overlooked the importance of allergens in causing the symptoms, or that the patient simply did not report the complete symptom information to doctor. More in-depth analysis is needed on this.

It has been reported that cow’s milk and egg white sensitization in children could have a high prevalence of around 41–67% [13]. Our findings agree with these results. This indicates that a close relation might exist between the allergens and skin diseases. A study conducted in Taiwan on 931 children found that early sensitization to egg white proteins can increase the risk of eczema [14]. Although we have also found that children have a higher positive rate to cow’s milk (24.7%) and egg white (27.8%), sensitization to cockroach seemed to be more significant in adults with urticaria (34.0%), atopic dermatitis (29.3%), and eczema (25.7%). This is in accordance with other findings that show that although egg white and cow’s milk are the major allergens to induce eczema and uritcaria [15], the positive rate to the two allergens always fall below 3%. This could be explained by the fact that when the children ingest the food allergen at the infant stage, their digestive enzymes are not well formed or secreted; the incompletely digested allergens can easily enter the gastrointestinal tracts to induce the allergic symptoms. When the children grow older, their gastrointestinal tracts also develop, which eventually leads to milder symptoms, and in certain cases, completely outgrowth of the allergy to cow’s milk and egg white [16]. In order not to excessively exclude foods and cause nutrition imbalance, children allergic to cow’s milk and egg are advised to check for sIgE regularly to re-evaluate their allergic status.

Our study showed that adults tend to have higher sensitization rate to shrimp and crab in both coastal and inland areas in southern China, especially among those adults with urticaria. This is similar to the findings of a study conducted in the US that among 5529 patients [17], sensitization to crab was significantly higher in adults than in children (23.0% vs. 13.1%). We believe this may due to the difference in ingestion volume, as in China, parents tend to control what and how much their children eat. A lower ingestion volume might lead to a lower allergic impact on the children. Since adults also have a higher sensitization to cockroach, its cross-reactivity with shrimp and crab could play a role as well.

A novel finding of the current study was that cockroach allergen had a high positive rate in allergic adults [18], Gruchalla et al. [19] also found that there were 69% inner-city asthma children positive to cockroach by SPT. This is in contrast to other previous studies that showed that just house dust mite was the predominant allergen in southern China [10]. This discrepancy may be due to the fact that the patients recruited in our study were mainly from the basic clinics and smaller hospitals in southern China. Compared with previous studies based on samples from coastal areas and bigger hospitals [20], patients covered in our study generally had a lower living standard and the cockroaches (including *Periplaneta americana*) in the environment maybe reproduction quickly, this may reflect a genuine real-life pattern.

We also found that 79.1% the patients had co-sensitization to crab, cockroach, shrimp, and house dust mite. Optimal scaling analysis showed a close correlation among sIgE level against crab, shrimp and cockroach. Of the patients in our study population 5309 were simultaneously positive for crab, cockroach, shrimp, and house dust mite, while only 13 patients were positive for crab alone. The relatively rare incidence of single allergy to crab seems to indicate that patients with crab allergies may be co-sensitized to other allergens such as some cross-reactive protein (tropomyosin and arginine kinase), and should avoid them [21, 22].

The current study showed that the sensitization to mold in children increased from summer to autumn and reached its peak in October (6.2%). It is well known that the concentration of airborne fungal spores shows monthly variation. A study in Romania showed that the proportion of airborne *Cladosporium* and *Alternaria* fungal spores started to rise in June and reached its peak in September [23]. This is in agreement with our findings, and implies that mold allergy in children may correlate to the airborne mold concentration. According to some previous studies [24, 25], airborne *Cladosporium* and *Alternaria* could grow in patients with disrupted respiratory tract and thereby induce infection and allergy. It is recommended that children with respiratory diseases related to epithelium disruption should take the appropriate preventive measures during the susceptible seasons, such as wearing a mouth mask and/or reducing outdoor activities [26]. Nevertheless, as no similar trend was seen in adults, more studies and analysis are needed to identify the underlying reasons. In addition, the present study just focused on common allergens in large population, workplace allergens such as chemicals should also be taken seriously, we look forward to more research in this area in the future.

## Conclusion

Cockroach and house dust mite were the most important allergens in adults and children respectively in southern China. This was the first study to report a greater significance of cockroach compared to house dust mite in adults, especially among those with multiple allergic symptoms. As the prevalence of sensitization to crab, cockroach, house dust mite and shrimp were high and cross-reactivity might exist between these allergens, clinicians should take these into consideration when they formulate the strategies for allergen avoidance in patients.

## Additional file

**Additional file 1: Table S1.** Positive rates of allergens with fewer than 3000 cases detected in KMD. Test numbers and the positive rates for Phadiatop, d2 (*Dermatophagoides farinae*), f4 (*Triticum aestivum*), fx1 (Food mixes 1), w6 (*Artemisia vulgaris*), w1 (*Ambrosia elatior*), m3 (*Aspergillus fumigatus*), f14 (Soya bean), wx5 (Weed mixes 5), e1 (Cat dander), and m6 (*Alternaria alternata*).

## Data Availability

The data that support these findings are available on reasonable request from the corresponding author BS. Data are not publicly available due to concerns regarding research participant privacy.

## Abbreviations

sIgE: specific immunoglobulin E
KMD: KingMed Diagnostics Center
